# miR-423-5p mediates LINC00886 regulation of ovarian cancer aggressiveness and immune evasion via the TLR4/Myd88/NF-κB/PD-L1 pathway

**DOI:** 10.1186/s41065-025-00540-2

**Published:** 2025-09-25

**Authors:** Na Du, Xiaowen Zhang, Chao He, Zheng Zhang

**Affiliations:** 1 Department of Gynecology, Heping Hospital Affiliated to Changzhi Medical College, Changzhi, 046000 Shanxi China; 2 Department of Gynecology, Kongjiang Hospital of Yangpu District, Shanghai, China; 3 Department of Obstetrics and Gynecology, The First Hospital of Qiqihar, Qiqihar City, 161005 Heilongjiang Province China; 4 School of Clinical Medicine, Hubei College of Chinese Medicine, No.87, Xueyuan Road, Jingzhou District, Jingzhou City, 434020 Hubei Province China

**Keywords:** LINC00886, Ovarian cancer, miR-423-5p, TLR4, Cellular processes, immune escape

## Abstract

**Background:**

Ovarian cancer has poor treatment outcomes. This study aims to explore the clinical importance of LINC00886 and its effects on cancer cell behavior in ovarian cancer, potentially offering a new therapeutic target.

**Materials and methods:**

RT-qPCR was used to detect LINC00886 expression in ovarian cancer tissue, with analysis of clinicopathological data and prognosis based on LINC00886 expression levels. CCK-8, Traswell, and Annexin V-FITC/PI flow cytometry assays were used to evaluate the impact of molecular expression on cell viability, invasiveness, and apoptosis. RIP and dual luciferase reporter gene assays were used to validate interactions among miR-423-5p, LINC00886, and TLR4. Western blot analysis was conducted to investigate downstream signaling proteins, and ELISA was used to measure TNF-α and IFN-γ levels in cell co-culture.

**Results:**

LINC00886 is upregulated in ovarian cancer tissues and cell lines, and its high expression is associated with poor prognosis; downregulating LINC00886 inhibits cell viability and invasiveness while inducing apoptosis. miR-423-5p is downstream of LINC00886 and upstream of TLR4. Inhibiting miR-423-5p reverses the suppressive effects of LINC00886 downregulation on cancer cell behavior. Overexpressing TLR4 enhances cellular processes. Furthermore, downregulating LINC00886 reduces the expression of TLR4, Myd88, phosphorylated NF-κB p65, and PD-L1, while increasing TNF-α and IFN-γ levels and enhancing CD8 + T cell antitumor activity, thereby reducing tumor cell immune escape.

**Conclusions:**

LINC00886 drives ovarian cancer progression and immune escape through themiR-423-5p/TLR4/Myd88/NF-κB/PD-L1 axis, establishing its potential as both a prognostic biomarker and therapeutic target.

**Supplementary Information:**

The online version contains supplementary material available at 10.1186/s41065-025-00540-2.

## Background

Ovarian cancer poses a global threat to women’s health. Its early symptoms are often overlooked and misdiagnosed, leading to late-stage diagnoses for most patients [1]. The prognosis for ovarian cancer is generally poor, with advanced cases having a low five-year survival rate and a high recurrence risk. Follow-up treatment after recurrence is extremely challenging [2, 3]. The complex pathogenesis of ovarian cancer, involving multiple intertwined factors, makes it a difficult clinical problem. Despite recent advancements in targeted therapy and immunotherapy, their effectiveness still requires enhancement. Therefore, exploring the molecular mechanisms underlying its occurrence and development is crucial for improving patients’ prognosis and quality of life.

Long non-coding RNAs (lncRNAs) are critical regulators of gene expression, cell differentiation, development and disease progression [4, 5]. Several reports have indicated that lncRNAs can act as competitive endogenous RNAs (ceRNAs) by binding to microRNAs (miRNAs), which in turn regulate the expression of downstream target genes and alter the behavior of tumor cells [6, 7]. For instance, LINC00665 enhances FHDC1 expression in ovarian cancer by binding to and inhibiting miR-181a-5p, thus accelerating cancer development and presenting a potential new therapeutic target for ovarian cancer [8]. Similarly, LINC01123 supports ovarian cancer development by inhibiting miR-516b-5p and upregulating VEGFA expression [9]. LncRNA AC005224.4 and lncRNA HAS2-AS1 have also been reported to regulate ovarian cancer progression via miRNA sponging [10, 11]. These findings collectively highlight the critical role of lncRNA-miRNA networks in ovarian cancer pathogenesis.

LINC00886, a newly identified lncRNA, plays an active role in cancer genesis and development. Specifically, it regulates thyroid cancer through the PKR/eIF2α signaling pathway [12] and its suppression enhances malignant activity in laryngeal cancer [13]. Notably, LINC00886 also shows abnormal expression in granulocytes of infertile women with ovarian endometriosis [14], hinting at a potential link to ovarian cancer development. However, the specific mechanism by which LINC00886 functions in ovarian cancer remains unknown and warrants further investigation.

Another important class of non-coding RNAs, miRNAs, were discovered to play a pivotal part in the initiation and progression of tumors through their interaction with lnRNAs [15, 16]. Importantly, a study has highlighted that miR-423-5p expression is notably downregulated in ovarian cancer tissues, possessing the capacity to suppress cancer cell proliferation and invasion, thereby suggesting its potential as an oncogenic factor in ovarian cancer [17]. miR-423-5p has been demonstrated to exert significant influence in the development of various types of tumors, including prostate cancer [18], colorectal cancer [19], and gastric cancer [20], among others, exhibiting notable oncogenic effects. Furthermore, LINC00680 has been found to promote the progression of esophageal squamous carcinoma by targeting miR-423-5p [21], while lncRNA NR2F1-AS1 also stimulates the behavioral activity of papillary thyroid carcinoma cells by modulating its expression [22]. However, there remains a dearth of in-depth research on the interaction mechanism between miR-423-5p and LINC00886 in the context of ovarian cancer.

The aim of this study was to investigate the function of LINC00886 in ovarian cancer and its interaction with miR-423-5p and to elucidate their impact on the development and progression of ovarian cancer. The objective is to uncover fresh theoretical insights and experimental proof that can contribute to the treatment of ovarian cancer, and to foster the evolution of molecular targeted therapeutic approaches for this malignancy.

## Methods

### Collection of clinical specimens

Between 2017 and 2019, a total of 106 specimens of ovarian cancer tissues and their adjacent non-cancerous tissues (located more than 2 cm away from the cancerous tissues and confirmed as normal through pathological examination) were surgically obtained from patients at The First Hospital of Qiqihar. These specimens were promptly snap-frozen in liquid ammonia and stored in an -80 °C refrigerator following collection.

Inclusion Criteria:

1. Permission was secured from the patients or their family members.
2. The diagnosis of ovarian cancer was confirmed through pathological examination.
3. The patients were experiencing their first diagnosis and had not undergone any other form of treatment prior to admission.

Exclusion Criteria:

1. Patients under the age of 18.
2. Incomplete or missing clinical data.
3. Secondary ovarian cancer.
4. Presence of malignant tumors in other locations.
5. Autoimmune diseases.
6. Coexistence of mental health disorders.

The ethics protocol for the study underwent review and received approval from the Ethics Committee at The First Hospital of Qiqihar. For a period of 5 years, ovarian cancer patients underwent follow-up assessments through telephone calls or outpatient visits. The patients continued to be monitored until their death or until 2024, at which point their survival rates were evaluated.

### Cell culture and cell transfection

The immortalized human ovarian surface epithelial cell line IOSE-80 was selected as a non-malignant control due to its established physiological relevance in retaining primary ovarian epithelium characteristics, documented genetic stability without oncogenic mutations, and scientific validation as a standard comparator in ovarian cancer research [23], alongside malignant ovarian cancer cell lines (SKOV3, COC1, OVCAR-3, A2780; BeiNa Biotech, China), with all cell types cultured under identical conditions for direct comparability. Cryopreserved cells were thawed in a 37 °C water bath and immediately cultured in RPMI1640 medium (Pernoside, Wuhan, China) with the addition of 10% fetal bovine serum and double antibody and incubated (37 ℃, 5% CO_2_). The culture medium was observed and changed over time (once three days), and after the cells had grown to a certain level (80% fusion), trypsin digestion was used for passaging. Using the liposome transfection technique (Lipofectamine ™2000, Thermo Fisher, USA), the targeted vector was introduced into COC1 and A2780 cells that were good growth status. These cells were then incubated for 48 h.

### Quantitative realtime PCR (qRTPCR)

RNA was isolated from tissue specimens and cancer cells belonging to different treatment groups, employing TRIzol reagent (Solarbio Science & Technology Co., Ltd., Beijing, China). The purity of the extracted RNA was verified using a DU800 spectrophotometer (Beckman Coulter, Fullerton, CA, USA). Following this, the RNA was converted into cDNA using a reverse transcription kit (Beyotime Biotechnology Co., Ltd., Shanghai, China). Utilizing the cDNA as a template, amplification was executed on an ABI 7500 Prism real-time fluorescent quantitative PCR platform, with the SYBR Premix Ex Taq kit (Beyotime Biotech Co., Ltd., Shanghai, China). The PCR amplification protocol involved an initial denaturation at 50 °C for 5 min, followed by 95 °C for 3 min, and subsequently 40 cycles of denaturation at 95 °C for 10 s and annealing/extension at 60 °C for 30 s. For normalization, GAPDH was chosen as the internal control for both lncRNA and mRNA, whereas U6 was selected for miRNA. The relative expressions of the genes were quantified by analyzing their cycle threshold values using the 2^−ΔΔCt^ method (The specific primer sequence is shown in Table S1).

### Dual luciferase gene reporter assay

To investigate the interaction sites of miR-423-5p with both LINC00886 and TLR4, bioinformatics tools from the LncRNASNPv3 (https://gong_lab.hzau.edu.cn/lncrnasnp3#!/) and StarBase databases (http://starbase.sysu.edu.cn/) were utilized to explore. Based on the identified binding sequences (detailed in Supplementary Table S2), fragments of wild-type (WT) LINC00886, WT-TLR4, and mutant (MT) LINC00886, MT-TLR4 were designed. The WT plasmids contained intact miR-423-5p binding sites predicted in silico, while MT plasmids carried point mutations in the seed regions to disrupt miRNA binding. These fragments were amplified via PCR using mutation-specific primers, cloned into pmirGLO reporter vectors (Promega, USA), and verified by Sanger sequencing. Subsequently, the constructed vectors were co-transfected into COC1 and A2780 cell lines along with miR-mimics, miR-inhibitors, and negative controls. Following a 48-hour incubation period, luciferase activity was quantitatively assessed using the Dual-Luciferase Reporter Assay SystemPromega, USA).

### RNA Immunoprecipitation (RIP) assay

The presence of miR-423-5p, LINC00886, and TLR4 within immunoprecipitation complexes was assayed using the EZ-Magna RIP RNA-Binding Protein Immunoprecipitation Kit supplied by Millipore (USA). The Argonaute-2 (Ago2) antibody was selected for RIP assays as Ago2 is the core catalytic component of the RNA-induced silencing complex (RISC). Its binding to miRNAs or lncRNAs indicates direct incorporation into functional miRNA-mediated silencing complexes, validating physical interactions within the ceRNA network [24, 25]. For this purpose, COC1 and A2780 cells were lysed with RIP buffer. Following this, magnetic beads coated with either human Ago2 antibody (anti-Ago2) or a negative control IgG antibody (anti-IgG) were incubated with the lysed cell solution at a temperature maintained at 4 °C. Subsequently, the expression levels of these RNA species were quantitatively determined by RT-qPCR.

### Western blot analysis

COC1 and A2780 Cells from different treatment groups were collected and lysed in pre-chilled cell lysis buffer. Following centrifugation at 13,000 rpm for 10 min at 4 °C, the supernatants were collected for BCA protein quantification. Equal amounts of protein samples were mixed with sample buffer and boiled for 5 min. The samples were then separated by SDS-PAGE electrophoresis at 120 mV and subsequently transferred onto nitrocellulose membranes using a semi-dry transfer method. The membranes were blocked with 5% skimmed milk for 1 h at room condition, followed by overnight incubation at 4 °C with primary antibodies against: Microtubule-associated protein 1 light chain 3-II (LC3-II, 1:2000), Cleaved cysteine-aspartic acid protease 3 (cleaved-caspase-3, 1:2000) Toll-like receptor 4 (TLR4, 1: 2000), myeloid differentiation primary response gene 88 (Myd88, 1:1000), phosphorylated nuclear factor-kappa B P65 (p-NF-κB p65, 1:1000) and programmed death ligand 1 (PD-L1, 1:1000), all from Cell Signaling Technology, USA. After washing with PBS, the membranes were incubated with secondary antibodies (1:2000) for 1 h. Protein bands were visualized using an electrochemiluminescence system by ECL substrate reagent, with GAPDH serving as the internal control to evaluate the relative protein expression levels.

### Flow cytometry

Successfully transfected COC1 and A2780 cells were initially digested with trypsin. Afterward, the cells were washed and resuspended. Using the (Membrane Associated Protein V-Fluorescein Isothiocyanate/Propidium Iodide (AnnexinV-FITC/PI) kit (Beyotime Biotech Co., Ltd., Shanghai, China), the cells were double-stained with 5 µL each of FITC-Annexin V and PI, while maintaining a dark environment for 5 min. Apoptosis analysis was then conducted using a FACSCalibur flow cytometer (Becton-Dickinson), and the apoptosis rate was subsequently calculated based on the obtained data.

### CCK-8 assay

Cell viability was assessed utilizing the Cell Counting Kit-8 (CCK-8, Beyotime Biotech Co., Ltd., Shanghai, China). COC1 and A2780 cells were harvested via centrifugation, washed, and resuspended for enumeration (1 × 10^3^ cells per well) before being seeded into 96-well plates. Subsequent to seeding, CCK-8 reagent (administered post-washing) was introduced at 0, 24, 48, and 72 h. Following an additional 2-hour incubation period, the optical density (OD) was measured at a wavelength of 450 nm.

### Transwell assay

A 60 µL aliquot of diluted Matrigel gel was applied to the apical compartment of the transwell chamber and allowed to solidify within a 37 °C incubator. Post-transfection, COC1 and A2780 cells from respective groups were harvested and resuspended in serum-free medium. These resuspended cells were seeded into the upper chamber, while the lower chamber was supplemented with serum-containing medium. Following a 24-hour incubation period, non-invasive cells adhering to the bottom surface of the chamber were gently removed using a cotton swab. The invasive cells were then fixed with formaldehyde for 10 min and stained with crystal violet for 20 min. After thorough rinsing and drying with PBS, the stained cells were examined microscopically and photographed for enumeration.

### CD8 + T cell apoptosis rate determined by flow cytometry

Human peripheral blood-derived CD8 + T cells (Huizhi and Yuan, Beijing, China) were activated for 48 h using1 µg/ml anti-CD28 antibody, 1 µg/ml anti-CD28 antibody, and 10 ng/ml of interleukin-2 (IL-2). Activated cells were then purified using the EasySep™ Human CD8⁺ T Cell Enrichment Kit (STEMCELL Technologies, Canada). For co-culture, a Transwell system was employed: transfected COC1 cells were inoculated in the upper chamber, while purified CD8 + T cells were placed in the lower chamber. After 24 h of co-culture, CD8 + T cells were harvested and stained with FITC–Annexin V and propidium iodide (PI) using the Annexin V-FITC/PI Apoptosis Kit (Beyotime, China) for 15 min in the dark. Apoptosis rates were immediately quantified via flow cytometry (FACSCalibur, BD), defined as the combined percentage of Annexin V⁺/PI⁻ (early apoptotic) and Annexin V⁺/PI⁺ (late apoptotic) cells. To confirm post-culture CD8⁺ T cell purity (> 95%), parallel samples were stained with FITC–anti-human CD8 antibody and analyzed by flow cytometry.

### Quantification of TNF-α and IFN-γ by ELISA

After 24 h of co-culture, supernatants were collected from the bottom culture dishes for detection. The concentrations of Tumor Necrosis Factor-alpha (TNF-α) and Interferon-gamma (IFN-γ) were measured in the cell culture supernatants of co-cultured cell following the ELISA kit protocol (Beyotime Biotech Co., Ltd., Shanghai, China). Each well plate received 50 µL of sample and 50 µL of standard solution, which were then incubated at 37 ℃ for 60 min before being removed. Subsequently, 100 µL of horseradish peroxidase-conjugated antibody was added to each well and incubated at 37℃ for 30 min. The wells were washed, and a substrate reagent was introduced. After incubating for 15 min at 37℃ in the dark, the reaction was halted by adding a stop solution. An ELISA reader was used to measure the OD_450_, and the levels of TNF-α and IFN-γ were subsequently calculated.

### Bioinformatics analysis

miR-423-5p downstream target was predicted with the tools miRDB (https://mirdb.org/), MiRWalk (http://mirwalk.umm.uni-heidelberg.de/) and Starbase database (https://rnasysu.com/encori/panCancer.php). After initially filtering the results based on specified thresholds, a Venn diagram is employed to identify the overlapping targets. Subsequently, Gene Ontology (GO) analysis and Kyoto Encyclopedia of Genes and Genomes (KEGG) enrichment are conducted on the target genes obtained from this intersection.

### Statistical analysis

Statistical analysis was conducted utilizing SPSS version 25.0. Normally distributed variables were reported as mean ± standard deviation, with comparisons among multiple groups employing one-way ANOVA. Quantitative data were compared pairwise through the application of the independent Student’s t-test. An assessment of the relationship between LINC00886 expression and the clinicopathological features of breast cancer patients was conducted using the chi-square test. Correlations were evaluated by calculating Pearson’s correlation coefficient. The threshold for statistical significance was established at a *P*-value of less than 0.05.

## Results

### Clinical significance of LINC00886 expression in ovarian cancer

**Fig. 1:**
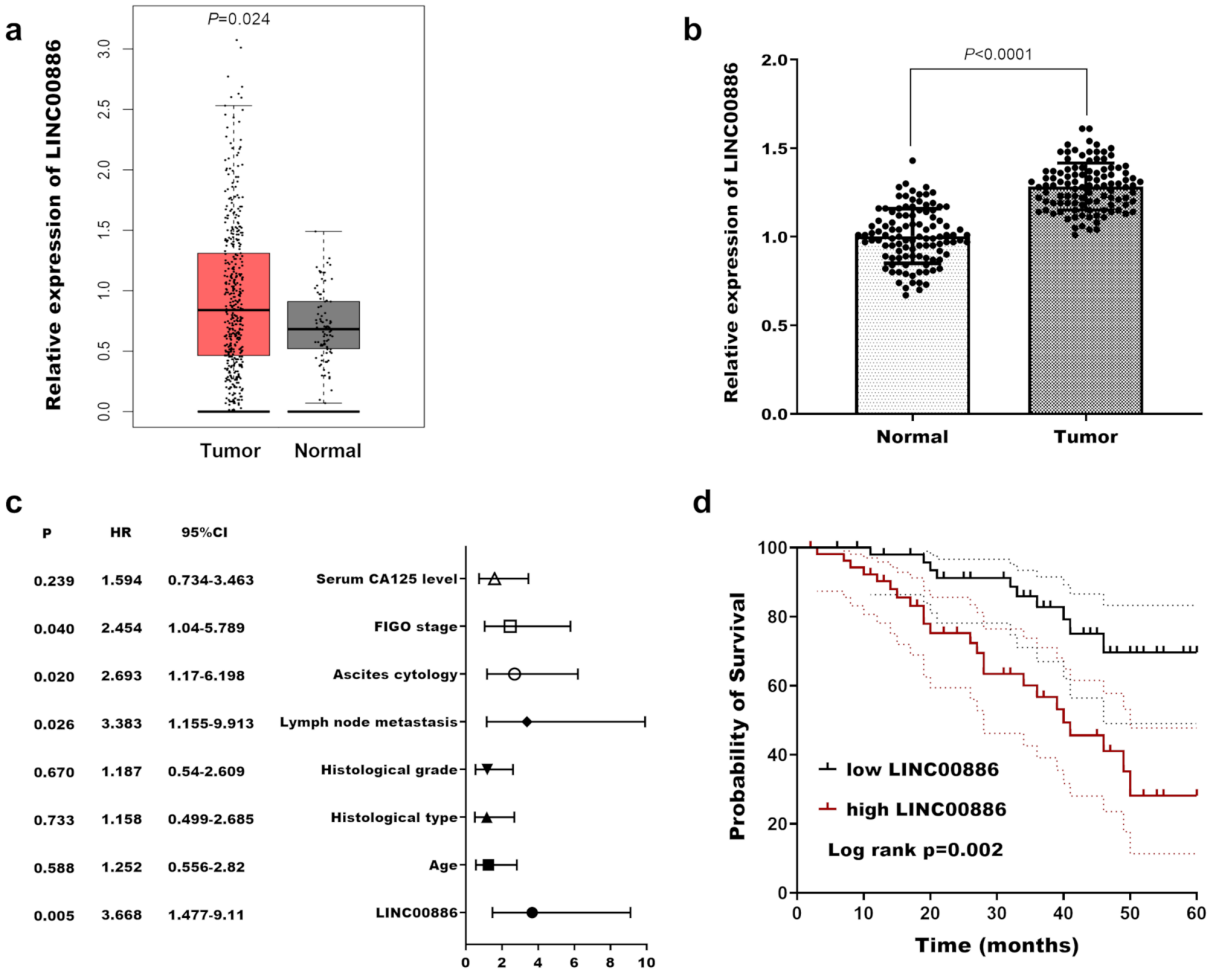
LINC00886 expression in ovarian cancer tissues and its correlation with clinical prognosis. (**a**) LINC00886 expression profiles in ovarian cancer tissues versus normal ovarian tissues from the GEPIA database (http://gepia.cancer-pku.cn/). Data presented as mean ± SEM. *P* = 0.024. (**b**) LINC00886 expression in 102 paired ovarian cancer tissues and adjacent non-cancerous tissues detected by quantitative real-time PCR (qRT-PCR). Data normalized to GAPDH and presented as mean ± SD. *P* < 0.001 by paired t-test. (**c**) Multivariate Cox regression analysis identifying prognosis factors for ovarian cancer. Hazard ratio (HR) with 95% confidence interval (CI) shown. Statistical significance determined by Wald test. (**d**) Kaplan-Meier survival curves illustrating overall survival differences between high (*n* = 54) and low (*n* = 52) LINC00886 expression groups, stratified by the mean expression value. Survival differences analyzed by log-rank test

Initial investigation of LINC00886 expression in tumors was conducted using the GEPIA database (http://gepia.cancer-pku.cn/index.html). As depicted in Fig. 1a, elevated LINC00886 expression was observed in ovarian cancer (OV, *P* = 0.024). Subsequent analysis of tissue samples from ovarian cancer patients further confirmed this significant upregulation (Fig. 1b, *P* < 0.0001). Comparison between patient groups with high and low LINC00886 expression revealed significant differences in lymph node metastasis, ascites cytology, and FIGO stage (*P* < 0.05; Table 1). FIGO stage refers to the International Federation of Gynecology and Obstetrics staging system for ovarian cancer, with stages I-II indicating early disease and III indicating advanced disease. Cox regression analyses identified LINC00886 (*P* = 0.007, HR = 3.624), lymph node metastasis (*P* = 0.026, HR = 3.383), ascites cytology (*P* = 0.020, HR = 2.693), and FIGO stage (*P* = 0.040, HR = 2.454) as prognostic factors for poor ovarian cancer outcomes (Fig. 1c). Additionally, Kaplan-Meier survival curves demonstrated that individuals with elevated LINC00886 expression had significantly shorter survival rates compared to those with lower expression levels (Log-rank *P* = 0.002; Fig. 1d).

**Table 1:** The association of LINC00886 with patients’ clinicopathological features

Variant	Cases (*n* = 106)	LINC00886 expression		*P*
		Low (*n* = 52)	High (*n* = 54)	
Age				0.166
< 60	48	20	28	
≥ 60	58	32	26	
Histological type				0.496
I	70	36	34	
II	36	16	20	
Histological grade				0.051
G1/G2	72	40	32	
G3	34	12	22	
Lymph node metastasis				0.042
Negative	76	42	34	
Positive	30	10	20	
Ascites cytology				0.047
Negative	74	41	33	
Positive	32	11	21	
FIGO stage				0.033
I-II	71	40	31	
III	35	12	23	
Serum CA125 level (U/ml)				0.079
< 500	73	40	33	
≥ 500	33	12	21	

### Effect of LINC00886 on the behavior of ovarian cancer cells

**Fig. 2:**
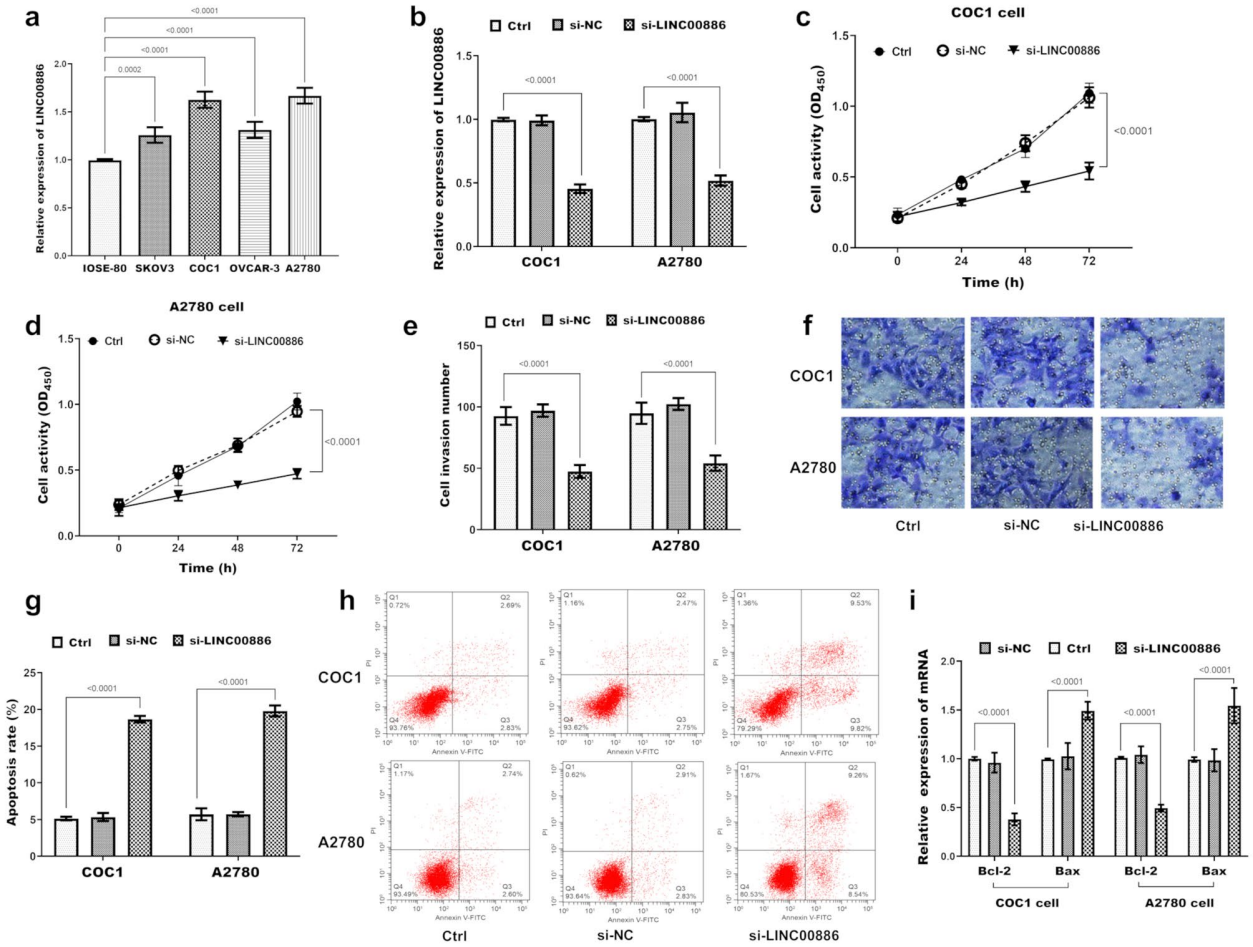
Regulatory effects of LINC00886 on biological behaviors of ovarian cancer cells. (**a**) LINC00886 expression levels in human ovarian cancer cell lines (COC1, A2780, SKOV3, OVCAR3) and normal ovarian epithelial cells (IOSE80), detected by qRT-PCR (GAPDH as internal reference, *n* = 3 independent experiments). *P* < 0.001 vs. IOSE-80 by one-way ANOVA. (**b**) Validation of LINC00886 downregulation efficiency in COC1 and A2780 cells 48 h after transfection with LINC00886-specific siRNAs (si-LINC00886) or negative control siRNA (si-NC), detected by qRT-PCR (*n* = 3). *P* < 0.0001 vs. si-NC by one-way ANOVA. (**c-d**) Cell viability of COC1 and A2780 cells transfected with si-LINC00886 or si-NC, measured by CCK-8 assay at 0, 24, 48, and 72 h (*n* = 5 replicates per time point). *P* < 0.0001 vs. si-NC by one-way ANOVA with Tukey’s post hoc test. (**e**) Quantitative analysis of cell invasiveness in COC1 and A2780 cells 48 h post-transfection with si-LINC00886 or si-NC, assessed by Transwell assay (Matrigel-coated). Invasive cells were stained with crystal violet, counted under a microscope (5 fields per well), and presented as the mean number of invasive cells per field (*n* = 3). *P* < 0.0001 vs. si-NC by one-way ANOVA with Tukey’s post hoc test. (**f**) Representative microscopic images of invasive cells (crystal violet-stained) from the Transwell assay, corresponding to the quantitative data in (**e**). (**g**) Quantitative analysis of apoptosis rates in COC1 and A2780 cells 48 h post-transfection with si-LINC00886 or si-NC, determined by Annexin V-FITC/PI flow cytometry. Data are presented as the total apoptotic rate (early apoptotic [Annexin V⁺/PI⁻] + late apoptotic [Annexin V⁺/PI⁺] cells, *n* = 3). *P* < 0.0001 vs. si-NC by one-way ANOVA with Tukey’s post hoc test. (**h**) Representative flow cytometry dot plots showing apoptotic cell populations (quadrant analysis: viable [Annexin V⁻/PI⁻], early apoptotic [Annexin V⁺/PI⁻], late apoptotic [Annexin V⁺/PI⁺], and necrotic [Annexin V⁻/PI⁺] cells) corresponding to the quantitative data in (**g**). (**i**) mRNA expression levels of pro-apoptotic Bax and anti-apoptotic Bcl-2 in COC1 and A2780 cells after LINC00886 knockdown, measured by qRT-PCR. Data were normalized to GAPDH and presented as fold-change vs. si-NC (*n* = 3). *P* < 0.0001 by one-way ANOVA with Tukey’s post hoc test

At the cellular level, analysis revealed significant upregulation of LINC00886 in four ovarian cancer cell lines (SKOV3, COC1, OVCAR-3, and A2780) compared to IOSE-80 cells (Fig. 2a, *P* *=* 0.0002, *P* < 0.0001). Transfection with si-LINC00886 significantly downregulated LINC00886 expression in COC1 and A2780 cells (Fig. 2b, *P* < 0.0001). This downregulation resulted in decreased viability (Fig. 2c, d, *P* < 0.0001) and invasiveness (*P* < 0.0001, Fig. 2e; representative images of cell invasion are shown in Fig. 2f.) and induced apoptosis (Fig. 2g and h provides flow cytometric visualization of apoptosis using Annexin V-FITC/PI dual staining) in both cancer cell lines. qRT-PCR analysis revealed that LINC00886 knockdown significantly increased mRNA expression of the pro-apoptotic marker Bax while suppressing mRNA levels of the anti-apoptotic marker Bcl-2 in both COC1 and A2780 cells (*P* < 0.0001; Fig. 2i).

### Interaction of miR-423-5p with LINC00886 and its effect on ovarian cancer cell behavior

**Fig. 3:**
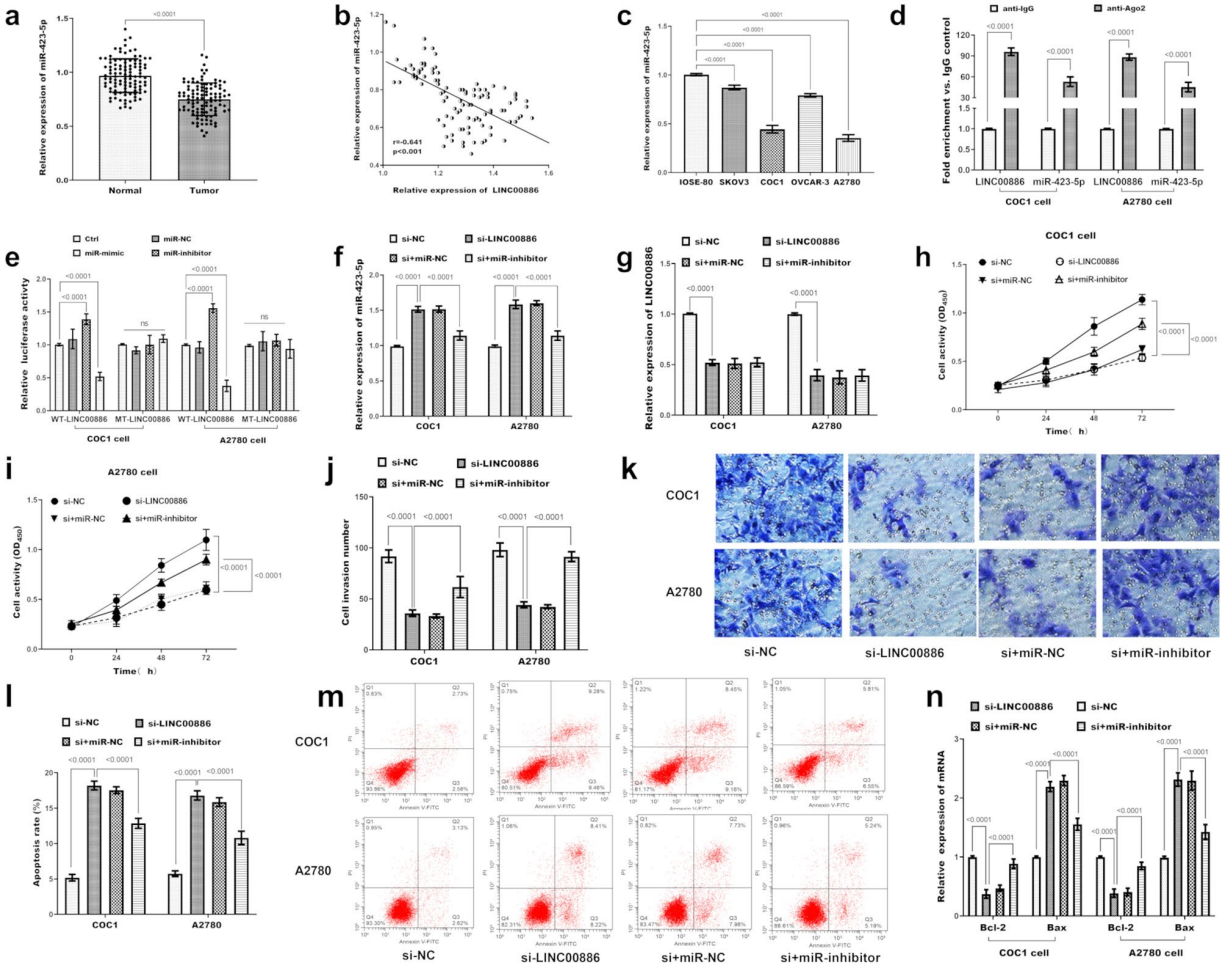
Interaction between miR-423-5p and LINC00886 and their impact on ovarian cancer cells. RT-qPCR analysis of miR-423-5p in clinical specimens normalized to U6. *P* < 0.0001 by paired t-test. (**b**) Correlation analysis between miR-423-5p and LINC00886 expression in ovarian cancer tissues, analyzed by Pearson’s correlation coefficient. (*r* = -0.641, *P* < 0.001). (**c**) miR-423-5p expression in ovarian cancer cell lines (COC1, A2780, SKOV3, OVCAR3) and normal ovarian epithelial cells (IOSE80), detected by qRT-PCR (U6 as internal reference, *n* = 3). *P* < 0.0001 vs. IOSE-80 by one-way ANOVA. (**d**) RNA immunoprecipitation (RIP) assay to validate the interaction between LINC00886 and miR-423-5p within the RNA-induced silencing complex (RISC). Argonaute 2 (Ago2), a core component of RISC that mediates miRNA-target RNA binding, was targeted using an anti-Ago2 antibody; IgG served as a negative control. Co-precipitated LINC00886 and miR-423-5p were detected by qRT-PCR in COC1 cells, confirming their co-localization in functional RISC (*n* = 3). This assay was designed to verify whether their interaction occurs in the context of active miRNA-mediated regulatory complexes. Data are mean ± SD. *P* < 0.001 (one-way ANOVA with Tukey’s post hoc test). (**e**) Dual-luciferase reporter assay to confirm sequence-specific binding between miR-423-5p and LINC00886. The wild-type (WT) LINC00886 reporter plasmid contains a fragment of LINC00886 harboring the predicted miR-423-5p binding site, while the mutant (MT) reporter plasmid carries site-directed mutations in the seed region of this binding site to disrupt specific interaction (both inserted into the pmirGLO vector, validated by Sanger sequencing). COC1/A2780 cells were co-transfected with WT/MT reporter plasmids and miR-423-5p mimics, miR-423-5p inhibitors or mimic NC; relative luciferase activity (firefly/renilla) was measured 48 h post-transfection (*n* = 3). The assay aims to demonstrate that miR-423-5p specifically binds to the predicted site in LINC00886, as reduced activity in WT + mimics (vs. mimic NC) and abrogated effect in MT groups would confirm sequence dependency. Data are mean ± SD. *P* < 0.0001 (one-way ANOVA with Tukey’s post hoc test). (**f**) miR-423-5p expression in COC1/A2780 cells after LINC00886 knockdown (si-LINC00886), detected by qRT-PCR (U6 as internal reference, *n* = 3). Data are mean ± SD. *P* < 0.001 (one-way ANOVA with Tukey’s post hoc test). (**g**) LINC00886 expression in COC1/A2780 cells after miR-423-5p knockdown (inhibitor), detected by qRT-PCR (GAPDH as internal reference, *n* = 3). Data are mean ± SD. *P* < 0.001 (one-way ANOVA with Tukey’s post hoc test). (**h-i**) Cell viability, (**j-k**) invasiveness, (**l-m**) apoptosis rate, and (**n**) Bax/Bcl-2 mRNA levels in COC1/A2780 cells after miR-423-5p knockdown, detected by CCK-8, Transwell, Annexin V-FITC/PI flow cytometry, and qRT-PCR, respectively (*n* = 3). Data are mean ± SD. *P* < 0.0001 (one-way ANOVA with Tukey’s post hoc test)

The expression of miR-423-5p in ovarian cancer tissues revealed significant downregulation (Fig. 3a, *P* < 0.0001), which exhibited a negative correlation with LINC00886 expression (*r* = -0.641, *P* < 0.001; Fig. 3b). Consistent with this, miR-423-5p was also found to be significantly decreased in ovarian cancer cell lines (Fig. 3c, *P* < 0.0001). Ago2 is a core component of the RISC, which mediates miRNA-target RNA interactions. To verify whether LINC00886 and miR-423-5p interact within functional RISC, we performed RIP assays using an anti-Ago2 antibody. Results showed significant co-precipitation of both LINC00886 and miR-423-5p in Ago2 immunoprecipitates compared to IgG controls (*P* < 0.0001, Fig. 3d), confirming their direct binding within the RISC complex. Dual luciferase reporter gene analysis demonstrated that upregulation of miR-423-5p significantly reduced luciferase activity in cells transfected with WT-LINC00886, but not in those with MT-LINC00886 (*P* < 0.0001, Fig. 3e). This confirms sequence-specific binding between miR-423-5p and LINC00886, as the mutated binding site (MT) abrogated the regulatory effect—ruling out non-specific interactions. Knockdown of LINC00886 significantly increased miR-423-5p expression (Fig. 3f), consistent with a ceRNA (competing endogenous RNA) model where LINC00886 sequesters miR-423-5p, and its depletion releases free miR-423-5p. Conversely, miR-423-5p knockdown did not alter LINC00886 levels (Fig. 3g), as ceRNA interactions typically involve miRNA sequestration rather than target degradation—supporting that LINC00886 regulates miR-423-5p, but not vice versa. miR-423-5p knockdown reversed the impact of LINC00886 downregulation on cell viability (Fig. 3h-i), invasion (Fig. 3j-k), and apoptosis (Fig. 3l-m), as well as the apoptosis-related proteins mRNA expression (Fig. 3n) in both cancer cell lines.

### Relationship between TLR4 and miR-423-5p

**Fig. 4:**
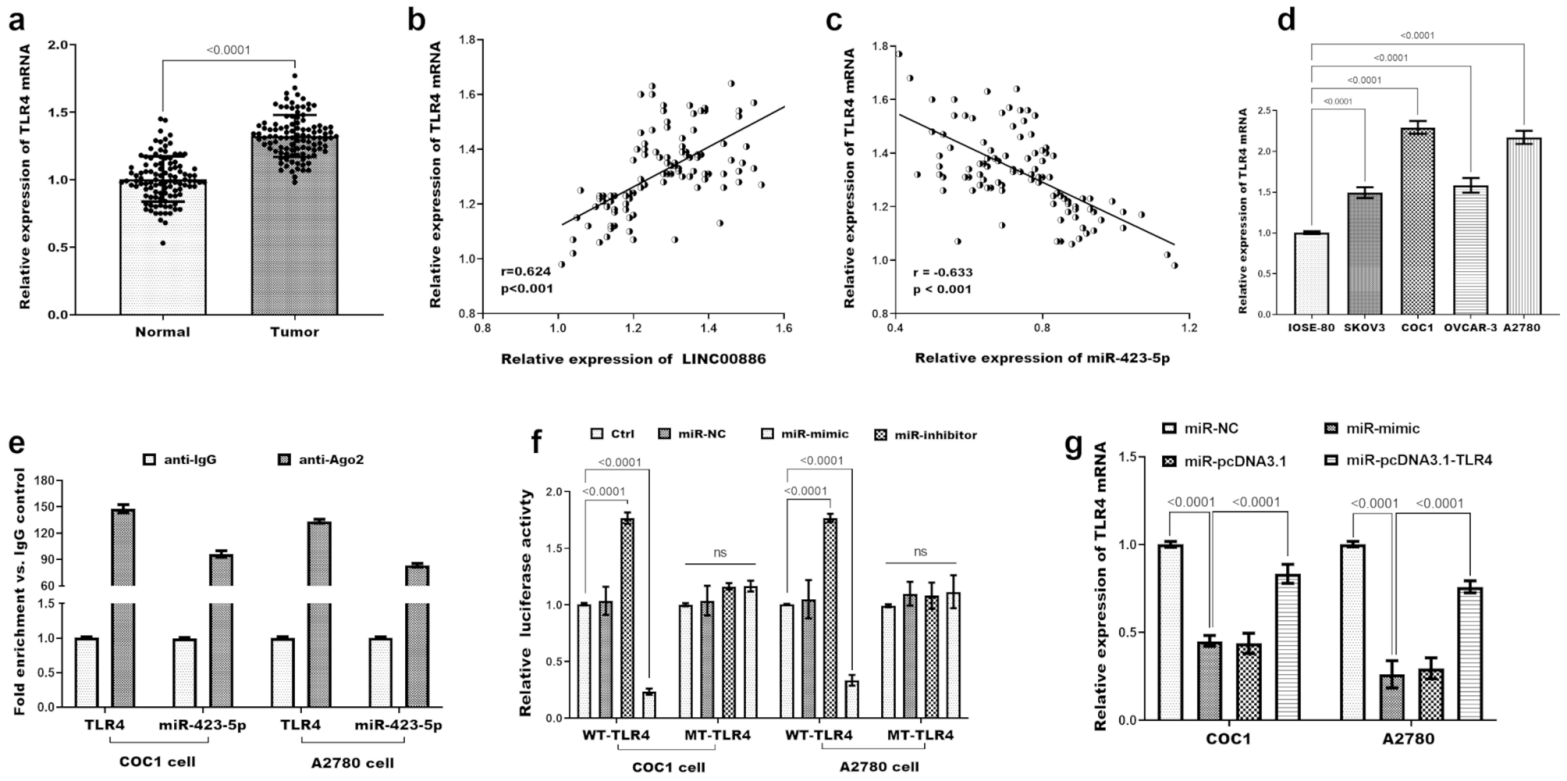
TLR4 expression in ovarian cancer and its regulatory relationship with miR-423-5p. (**a**) TLR4 mRNA expression in paired ovarian cancer tissues and adjacent non-cancerous tissues, detected by qRT-PCR (GAPDH as reference). Data are mean ± SD. *P* < 0.0001 (paired t-test). (**b-c**) Pearson’s correlation analysis between TLR4 and LINC00886 (b, r = 0.624, *P* < 0.001)/miR-423-5p (c, r = -0.633, *P* < 0.001) expression in ovarian cancer tissues. (**d**) TLR4 mRNA expression in ovarian cancer cell lines and IOSE80 cells, detected by qRT-PCR (GAPDH as reference, n = 3). Data are mean ± SD. *P* < 0.0001 (one-way ANOVA). (**e**) RIP assay using anti-Ago2 antibody in COC1 and A2780 cells, showing co-precipitation of TLR4 mRNA and miR-423-5p (qRT-PCR detection, IgG as control, n = 3). Data are mean ± SD. *P* < 0.0001 (one-way ANOVA with Tukey’s post hoc test). (**f**) Dual-luciferase reporter assay: COC1/A2780 cells co-transfected with WT/MT TLR4 3’-UTR reporter plasmid and miR-423-5p mimics/mimic NC; relative luciferase activity measured 48 h post-transfection (*n* = 3). Data are mean ± SD. *P* < 0.0001 (one-way ANOVA with Tukey’s post hoc test). (**g**) TLR4 mRNA expression in COC1/A2780 cells 48 h after transfection with miR-423-5p mimics or mimic NC, detected by qRT-PCR (*n* = 3). Data are mean ± SD. *P* < 0.0001 (one-way ANOVA with Tukey’s post hoc test)

As depicted in Figure S1a, an intersection analysis of predictions from three databases led to the identification of 120 target genes for miR-423-5p. Subsequent GO analysis (Figure S1b) highlighted enrichment of these target genes in processes such as cell morphogenesis regulation (BP), Ras protein signal transduction (BP), focal adhesion (CC), protein serine/threonine kinase activity (MF), and dependent protein binding (MF), among others, which are linked to cancer cell initiation and progression. KEGG pathway analysis presented in Figure S1c demonstrated a significant enrichment of these target genes in pathways including Focal adhesion, Proteoglycans in cancer, and MAPK signaling pathway, among others, all pertinent to cancer cell development. As one of the target genes, TLR4 was selected for further exploration due to its significant role in immune escape. TLR4 expression was markedly elevated in ovarian cancer tissues compared to normal tissues (Fig. 4a). In ovarian cancer tissues, a positive linear correlation was detected between TLR4 and LINC00886 (r = 0.624, *P* < 0.001; Fig. 4b). In contrast, a negative association was found between TLR4 and miR-423-5p (r = -0.633, *P* < 0.001; Fig. 4c). TLR4 was significantly raised in ovarian cancer cell lines (Fig. 4d). RIP assays confirmed that TLR4 and miR-423-5p co-precipitate in RISC complexes (Fig. 4e), indicating their direct interaction. Dual luciferase reporter further validated this: in Fig. 4f, luciferase activity of the WT-TLR4 3’-UTR reporter was significantly reduced by miR-423-5p mimics compared to miR-NC (*P* < 0.001), while no change was observed for the MT-TLR4 reporter (with mutated binding sites), confirming sequence-specific binding. Transfection with miR-423-5p mimics significantly suppressed TLR4 mRNA expression in two ovarian cancer cell lines (Fig. 4g).

### TLR4 overexpression partially reverses miR-423-5p-mediated biological effects

**Fig. 5:**
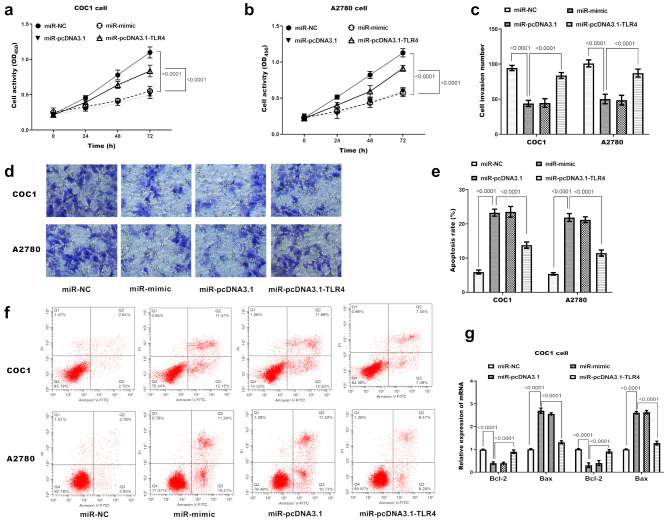
TLR4 mediates the regulatory effects of miR-423-5p on biological behaviors of ovarian cancer cells. (**a-b**) Cell viability measured by CCK-8 assay. COC1 and A2780 cells were transfected with miR-NC (negative control for miR-423-5p mimics), miR-mimic (miR-423-5p overexpression), miR-mimics + pcDNA3.1 (co-transfection of miR-423-5p mimics and empty pcDNA3.1 vector) or miR-mimics + pcDNA3.1-TLR4 (co-transfection of miR-423-5p mimics and TLR4 overexpression plasmid). Optical density (OD450) was measured at 0, 24, 48, and 72 h. Data = mean ± SD (*n* = 5 wells/group). *P* < 0.0001 vs. miR-NC; *P* < 0.0001 vs. miR-mimic (two-way ANOVA with Tukey’s post hoc test). (**c**) Quantification of invaded cells from Transwell assays. Cells in 5 random fields/well counted. Data = mean ± SD (*n* = 3). *P* < 0.0001 vs. miR-NC; *P* < 0.0001 vs. miR-423-5p mimic (one-way ANOVA, Tukey’s). (**d**) Representative crystal violet-stained images of invaded cells from Transwell invasion assays. (**e**) Apoptosis rates quantified by Annexin V-FITC/PI flow cytometry. Apoptotic cells = Annexin V⁺/PI⁻ + Annexin V⁺/PI⁺ populations. Data = mean ± SD (*n* = 3). *P* < 0.0001 vs. miR-NC; *P* < 0.0001 vs. miR-mimic (one-way ANOVA, Tukey’s post hoc). (**f**) Representative Annexin V/PI flow cytometry dot plots from three independent experiments. (**g**) mRNA expression of Bax and Bcl-2 detected by RT-qPCR. Total RNA reverse-transcribed to cDNA; amplification performed with SYBR Green. Data normalized to GAPDH = mean ± SD (*n* = 3). *P* < 0.0001 vs. miR-NC; *P* < 0.0001 vs. miR-mimic (one-way ANOVA, Tukey’s post hoc)

TLR4 overexpression only partially rescued miR-423-5p-mediated suppression of viability (Fig. 5a-b) and invasion (Fig. 5c-d), and similarly attenuated—but did not abolish—miR-423-5p-induced apoptosis (Fig. 5e). The partial reversal of miR-423-5p’s pro-apoptotic effect by TLR4 overexpression is attributed to a dual targeting mechanism: Transfection with miR-423-5p mimics alone significantly increased apoptosis by inhibiting endogenous TLR4 (*P* < 0.0001 vs. miR-NC). However, co-transfection of miR-423-5p mimics with the TLR4 plasmid (miR-pcDNA3.1-TLR4) failed to fully restore anti-apoptotic signaling. This is because TLR4 overexpression in the presence of miR-423-5p can only partially restore TLR4 protein levels (due to concurrent miR-423-5p-mediated suppression), resulting in incomplete rescue of TLR4’s anti-apoptotic function. Thus, the residual pro-apoptotic effect of miR-423-5p (unabated by partial TLR4 recovery) explains why apoptosis in the miR-pcDNA3.1-TLR4 group remains substantially higher than in miR-NC. Consistent with this, Fig. 5f provides direct visualizations of apoptotic cells across groups, and Fig. 5g shows corresponding changes in apoptosis-related markers, collectively validating this regulatory pattern.

### TLR4 downstream signaling pathway and its effect on tumor cell immune escape

**Fig. 6:**
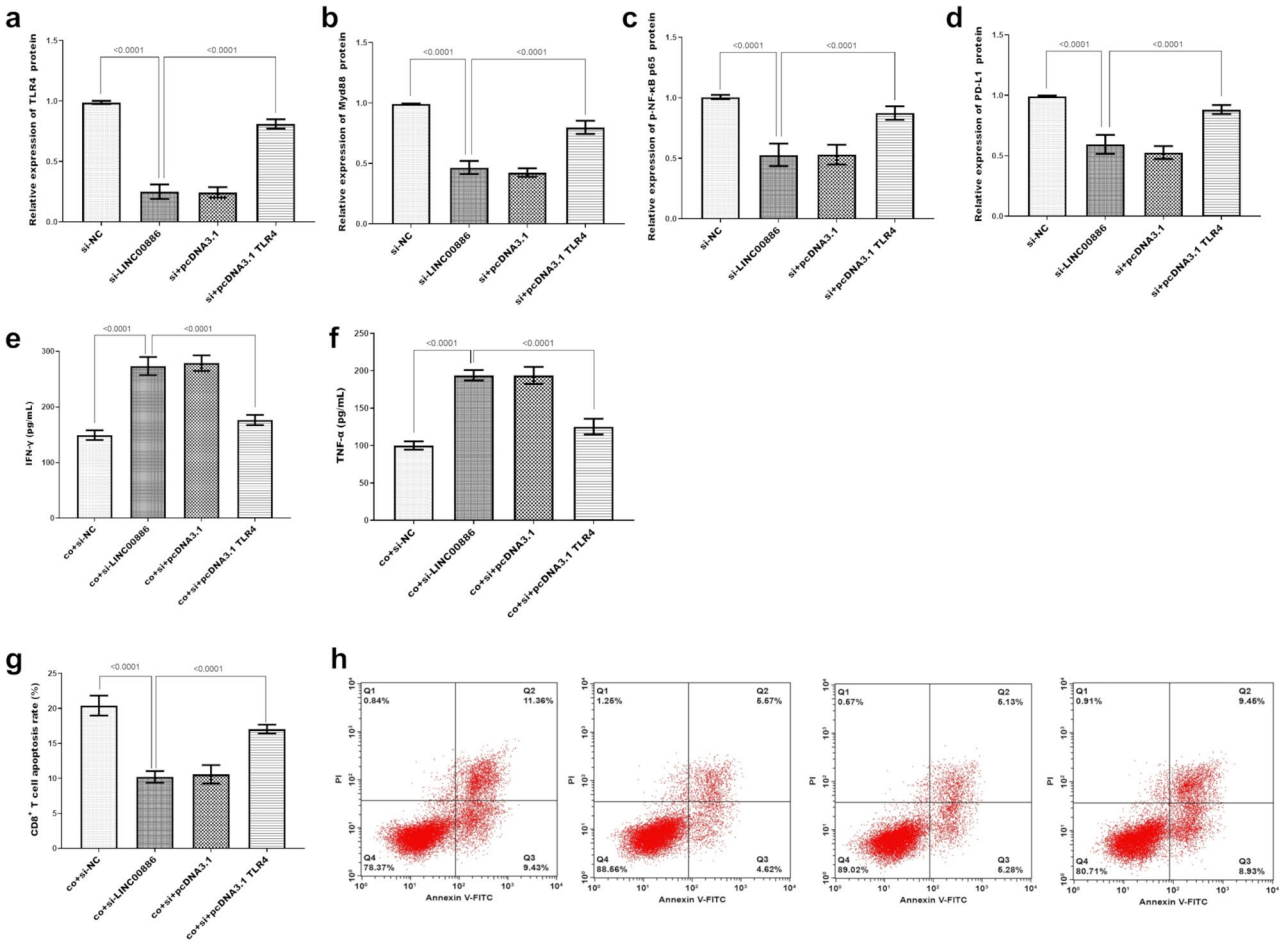
Regulation of LINC00886 on TLR4 signaling pathway and ovarian cancer immune escape. (**a-d**) Quantitative analysis of protein expression levels of TLR4 (**a**), Myd88 (**b**), phosphorylated NF-κB p65 (p-NF-κB p65, (**c**), and PD-L1 (**d**) in COC1 cells transfected with: (1) si-NC (negative control for LINC00886 knockdown); (2) si-LINC00886 (LINC00886 knockdown); (3) si + pcDNA3.1 (co-transfection of LINC00886 siRNA and empty pcDNA3.1 vector); (4) si + pcDNA3.1-TLR4 (co-transfection of LINC00886 siRNA and TLR4 overexpression plasmid). Proteins were detected by Western blot 48 h post-transfection. Band intensities quantified by ImageJ and normalized to GAPDH. Data = mean ± SD (*n* = 3). *P* < 0.0001 vs. si-NC; *P* < 0.0001 vs. si-LINC00886 (one-way ANOVA, Tukey’s post hoc). (**e-f**) Levels of TNF-α (**e**) and IFN-γ (**f**) in supernatants from transwell co-culture systems (CD8⁺ T cells in lower chamber; transfected COC1 cells in upper chamber, separated by a porous membrane) 48 h post-co-culture. Cytokine concentrations were measured by sandwich ELISA, with standard curves used for quantification (*n* = 3). Data = mean ± SD (*n* = 3). *P* < 0.0001 vs. co + si-NC;*P* < 0.001 vs. co + si-LINC00886 by one-way ANOVA. (**g**) Quantitative analysis of CD8⁺ T cell apoptosis rates in the co-culture system, determined by Annexin V-FITC/PI double staining and flow cytometry. Apoptosis rate is defined as the percentage of early apoptotic (Annexin V⁺/PI⁻) and late apoptotic (Annexin V⁺/PI⁺) cells (*n* = 3). (**h**) Representative flow cytometry dot plots showing apoptotic CD8⁺ T cell populations (quadrants: viable [Annexin V⁻/PI⁻], early apoptotic [Annexin V⁺/PI⁻], late apoptotic [Annexin V⁺/PI⁺], necrotic [Annexin V⁻/PI⁺]) corresponding to the quantitative data in (**g**). Data = mean ± SD (*n* = 3). *P* < 0.0001 vs. co + si-NC; *P* < 0.001 vs. co + si-LINC00886 by one-way ANOVA

In an in-depth exploration of the downstream signaling pathway employing COC1 cells as a model, Western blot analysis revealed that LINC00886 knockdown significantly suppressive protein levels of TLR4, Myd88, p NF-κB p65, and PD-L1, as quantified in Fig. 6a-d, while overexpression of TLR4 significantly reversed the situation. Representative immunoblots are provided in Supplementary Figure S2. TNF-α and IFN-γ levels in Fig. 6e and f were measured in cell culture supernatants from a transwell co-culture system, where CD8⁺ T cells were seeded in the lower chamber and transfected COC1 cells in the upper chamber (separated by a porous membrane to permit exchange of soluble factors while preventing direct cell contact). Supernatants were collected after the co-culture period, and cytokine levels were quantified using ELISA. Figure 6g shows quantitative data of CD8⁺ T cell apoptosis rates (mean ± SD, *n* = 3) from three independent experiments, and Fig. 6h (visual apoptotic images) includes representative results from these experiments, confirming consistent trends across all replicates. Analysis of the co-culture system revealed that TNF-α (Fig. 6e) and IFN-γ levels (Fig. 6f) were markedly higher in the LINC00886 knockdown group, while the apoptosis rate of CD8 + T cells was significantly lower (Fig. 6g-f). Overexpression of TLR4 significantly enhances TLR4/Myd88/NF-κB/PD-L1 signaling and the apoptosis of CD8 + T cells, thereby facilitating tumor cell immune escape and reversing the regulatory effects induced by LINC00886 downregulation.

### LINC00886 modulates autophagy and apoptosis through the miR-423-5p/TLR4 axis

To investigate the involvement of autophagy and apoptosis in the regulatory network of LINC00886 and miR-423-5p, we detected key molecular markers using Western blot. As shown in Supplementary Figure S3a, knockdown of LINC00886 led to a significant reduction in the expression of LC3-II (a canonical marker of autophagosome formation, indicative of autophagic activity) and cleaved-caspase-3 (a definitive marker of apoptotic activation), suggesting that depletion of LINC00886 simultaneously suppresses both autophagy and apoptosis. To determine whether miR-423-5p mediates these effects, we performed co-knockdown of LINC00886 and miR-423-5p. Results in Supplementary Figure S3b demonstrated that co-knockdown of miR-423-5p reversed the inhibitory effects of LINC00886 knockdown: levels of LC3-II and cleaved-caspase-3 were significantly restored, indicating that miR-423-5p is a critical mediator of LINC00886’s regulation of both autophagy and apoptosis. We further validated the downstream signaling by examining the role of TLR4, a known target of miR-423-5p. Supplementary Figure S3c showed that overexpression of TLR4 significantly reversed the promotion of LC3-II and cleaved-caspase-3 expression induced by miR-423-5p overexpression. These data collectively support a LINC00886/miR-423-5p/TLR4 regulatory cascade that modulates autophagy and apoptosis.

## Discussion

In recent years, increasing attention has been directed towards the role of lncRNA in the initiation and development of ovarian cancer. This study has preliminarily identified LINC00886 as a upregulated lncRNA in ovarian cancer, with its abnormal expression closely associated with unfavorable prognosis. Besides, it has been further unveiled that LINC00886 impacts the malignant progression and immune evasion of ovarian cancer by modulating miR-423-5p and the downstream TLR4 signaling pathway, offering a fresh perspective for therapeutic interventions in ovarian cancer.

This research first explored the significance of LINC00886 in ovarian cancer, revealing its elevated expression in ovarian cancer tissues and a tight link to both aggressive tumor progression and adverse patient outcomes. Analogously, lncRNA PVT1 is also overexpressed in ovarian cancer tissues, associated with late-stage disease and a grim prognosis, while facilitating cancer cell growth by suppressing miR-214 [26]. Furthermore, LINC00261 has been identified as a marker for poor prognosis in ovarian cancer patients, regulating the malignant behavior of tumor cells through the sponging of miR-545-3p [27]. These discoveries imply that LINC00886 could function not merely as an indicator of malignant progression and poor prognosis in ovarian cancer, but also as a pivotal component within the gene regulatory networks underlying the pathogenesis of this disease.

Based on above, it explored the impact of LINC00886 expression on the malignant behaviors of ovarian cancer cells and verified that suppressing LINC00886 expression can effectively hinder tumor cell viability and trigger their apoptosis. In esophageal cancer, LINC00886 modulates the activity of cancer cells and the epithelial-mesenchymal transition process by regulating the miR-144 pathway [28]. Liu et al. also demonstrated that in hepatocellular carcinoma, LINC00886 activates the NF-κB pathway and upregulates RAB10 and E2F2 by decreasing the levels of miR-409-3p and miR-214-5p, thereby enhancing the malignant behavior of these cells [29]. This further highlights the critical role of LINC00886 as an oncogene in the malignant development of ovarian cancer cells. Further exploration of the molecular mechanism disclosed that miR-423-5p has a direct binding site with LINC00886. miR-423-5p was considerably down-regulated in ovarian cancer, and its expression was negatively regulated by LINC00886. Wang et al. also verified the decreased expression of miR-423-5p in ovarian cancer and showed that miR-423-5p exerts an oncogenic effect within ovarian cancer [30]. The downregulation of LINC00319 was able to curtail the malignant activity of ovarian cancer cells by repressing the expression of NACC1 through miR-423-5p [31]. In a similar vein, in this study, it was discovered that suppressing the expression of miR-423-5p counteracted the inhibitory impact of silencing LINC00886 on the malignant behavior of cancer cells. This suggests that LINC00886 might influence the biological characteristics of ovarian cancer cells by modulating miR-423-5p.

miR-423-5p emerges as a versatile regulator of multiple cell death and survival mechanisms beyond apoptosis. In the context of ferroptosis, it is targeted by LINC01606 (acting as a ceRNA), with LINC01606 upregulation suppressing ferroptosis via miR-423-5p downregulation, which in turn activates the Wnt/β-catenin pathway and enhances SCD1 expression [32]. For autophagy, Stiuso et al. (PMID:25782064) demonstrated its pro-autophagic role in cancer cells. In apoptosis, miR-423-5p modulates caspase-dependent pathways, as shown in colorectal cancer where it induces caspase-3, -8, -9, and p53 expression [33–35]. Together with our findings that miR-423-5p mediates LINC00886’s regulation of autophagy and apoptosis, these data highlight miR-423-5p as a key node integrating ferroptosis, autophagy, and apoptosis signaling, underscoring its broad role in cell fate control.

Further research indicated that miR-423-5p can specifically bind to and target TLR4. Previous studies have shown that inhibiting the upregulated TLR4 expression in ovarian cancer can suppress its proliferation and invasion [36, 37]. The same findings were observed in this study as well. Similarly, in colorectal cancer, restoring TLR4 expression was observed to reverse the inhibitory action of miR-5195-3p upregulation on cancer cell activity [38]. This was also the case in this study, where overexpression of TLR4 was a driver of malignant activity in ovarian cancer cells. Multiple studies have shown that TLR4 expression in the tumor microenvironment is tightly linked to the immune defense. Immune escape not only enables tumor cells to evade immune surveillance and clearance but also facilitates sustained tumor growth, invasion, and metastasis [39, 40]. In non-small cell lung cancer, the activation of TLR4 has been shown to promote the expression of PD-L1. This mechanism creates a favorable environment for immune escape by inhibiting the activity of cytotoxic T lymphocytes, thereby significantly promoting tumor progression [41]. Further research by Youn et al. revealed the mechanism by which TLR4 upregulates PD-L1 expression in pancreatic cancer cells through the MyD88/NF-κB pathway, and pointed out that blocking the signaling of this pathway can, to a certain extent, inhibit the immune escape process of tumor cells [42]. Our study discovered that knocking out LINC00886 not only effectively inhibits the expression of TLR4, Myd88, NF-κB, and PD-L1 proteins but also significantly obstructs the immune escape process. Specifically, this is manifested by increased secretion of TNF-α and IFN-γ, as well as a marked elevation in the proportion of CD8 + T cells. These changes indicate a significant enhancement of the body’s antitumor immune response, which aids in more effectively recognizing and eliminating tumor cells [43, 44]. Immune escape is closely linked to disease progression and prognosis in cancer patients. Once tumor cells undergo immune escape, they are prone to malignancy and can metastasize to distant organs and tissues, leading to rapid deterioration of the condition and a significant reduction in patient survival rates. Considering the correlation between LINC00886 and lymph node metastasis, FIGO staging, and patient prognosis, we preliminarily speculate that LINC00886 may regulate immune escape in ovarian cancer through the miR-423-5p/TLR4/Myd88/NF-κB/PD-L1 axis, thereby promoting the malignant development of tumors and influencing patients’ survival outcomes.

However, several critical areas still require deeper investigation. Specifically, the comprehensive understanding of how LINC00886 interacts with other molecules or pathways to influence ovarian cancer progression and immune evasion is incomplete. Moreover, practical hurdles, including achieving drug specificity, ensuring safety, and validating efficacy, must be tackled through meticulous animal studies. Future research will prioritize overcoming these challenges to achieve significant breakthroughs in ovarian cancer treatment.

## Conclusions

In brief, this study uncovered the potential of LINC00886 in predicting poor prognosis in ovarian cancer, along with its underlying molecular mechanism. This mechanism may influence the malignant behavior and immune escape of ovarian cancer cells by modulating the miR-423-5p/TLR4/Myd88/NF-κB/PD-L1 pathway.

## Supplementary Information

Below is the link to the electronic supplementary material.

Supplementary Figure 1: Prediction of miR-423-5p target genes and their functional enrichment analysis (**a**) Venn diagram showing overlapping target genes of miR-423-5p identified by three miRNA target prediction databases (TargetScan, miRDB, and StarBase). Intersection analysis of predictions from these databases yielded 120 unique target genes, which were selected for subsequent functional enrichment analysis. (**b**) Gene Ontology (GO) functional enrichment analysis of the 120 overlapping target genes, performed using DAVID. The analysis includes three categories: biological process (BP), cellular component (CC), and molecular function (MF). Significantly enriched terms are displayed, including key processes such as “regulation of cell morphogenesis” (BP), “Ras protein signal transduction” (BP), “focal adhesion” (CC), “protein serine/threonine kinase activity” (MF), and “dependent protein binding” (MF), which are associated with cancer initiation and progression. (**c**) Kyoto Encyclopedia of Genes and Genomes (KEGG) pathway analysis of the 120 target genes, conducted using DAVID. The analysis revealed significant enrichment in pathways relevant to cancer development, including “Focal adhesion,” “Proteoglycans in cancer,” and “MAPK signaling pathway.” For both GO and KEGG analyses, enrichment significance was determined by Fisher’s exact test (*P* < 0.05), with the top enriched terms/pathways displayed

Supplementary Table 1: Primer sequences

Supplementary Table 2: Sequence of double luciferase reporter gene

Supplementary Figure 2: Full Western blot gel images for proteins in Fig. 6a-d. Full-length gel images of representative immunoblots from Western blot analysis, corresponding to the quantitative data in Fig. 6a-d. These complete gel images show protein bands for TLR4, Myd88, phosphorylated NF-κB p65 (p-NF-κB p65), PD-L1, and GAPDH (loading control) in COC1 cells under four transfection conditions: (1) si-NC (negative control siRNA); (2) si-LINC00886 (LINC00886 knockdown); (3) si + pcDNA3.1 (co-transfection of si-LINC00886 and empty pcDNA3.1 vector); (4) si-LINC00886 + pcDNA3.1-TLR4 (co-transfection of si-LINC00886 and TLR4 overexpression plasmid)

Supplementary Figure 3: Regulation of LINC00886/miR-423-5p/TLR4 axis on autophagy and apoptosis markers. (**a**) Protein expression levels of autophagy marker LC3-II (autophagosome marker) and apoptotic marker cleaved-caspase-3 in COC1 cells transfected with si-NC (negative control for LINC00886 knockdown) or si-LINC00886 (LINC00886 knockdown), detected by Western blot 48h post-transfection. (**b**) Protein levels of LC3-II and cleaved-caspase-3 in COC1 cells transfected with: (1) si-NC; (2) si-LINC00886; (3) si + miR-NC (co-knockdown of LINC00886 and negative control for miR-423-5p inhibitor); (4) si + miR-inhibitor (co-knockdown of LINC00886 and miR-423-5p inhibitor), detected by Western blot 48h post-transfection. (**c**) Protein levels of LC3-II and cleaved-caspase-3 in COC1 cells transfected with: (1) miR-NC (negative control for miR-423-5p mimics); (2) miR-mimic (miR-423-5p overexpression); (3) miR-mimics + pcDNA3.1 (co-transfection of miR-423-5p mimics and empty pcDNA3.1 vector); (4) miR-mimics + pcDNA3.1-TLR4 (co-transfection of miR-423-5p mimics and TLR4 overexpression plasmid), detected by Western blot 48h post-transfection. For all panels: relative protein expression was quantified by densitometry (ImageJ) and normalized to the control group; data are presented as mean ± SD (*n* = 3). Statistical analysis was performed using one-way ANOVA

## Data Availability

All data generated or analyzed during this study are included in this article. Further enquiries can be directed to the corresponding author.

## Abbreviations

ceRNAs: Competitive endogenous RNAs
GO: Gene Ontology
KEGG: Kyoto Encyclopedia of Genes and Genomes
lncRNAs: Long non-coding RNAs
OD: Optical density
qRT PCR: Quantitative Real time PCR
RIP: RNA immunoprecipitation
WT: Wild-type

## References

[1] Blanc-Durand F, Clemence Wei Xian L, Tan DSP. Targeting the immune microenvironment for ovarian cancer therapy. Front Immunol. 2023;14:1328651. 10.3389/fimmu.2023.1328651 38164130 10.3389/fimmu.2023.1328651 PMC10757966

[2] Salutari V, Giudice E, Lorusso D. Maintenance therapy for newly and recurrent epithelial ovarian cancer: current therapies and future perspectives. Curr Opin Obst Gynecol. 2024;36(1):9–17. 10.1097/gco.0000000000000931 38170548 10.1097/GCO.0000000000000931

[3] Wang Q, Cao SH, Li YY, Zhang JB, Yang XH, Zhang B. Advances in precision therapy of low-grade serous ovarian cancer: A review. Medicine. 2024;103(17):e34306. 10.1097/md.0000000000034306 38669365 10.1097/MD.0000000000034306 PMC11049748

[4] Yang S, Ji J, Wang M, Nie J, Wang S. Construction of ovarian cancer prognostic model based on the investigation of Ferroptosis-Related LncRNA. Biomolecules. 2023;13(2). 10.3390/biom13020306 10.3390/biom13020306 PMC9953467 36830675

[5] Shen W, Xie X, Liu M, Wang L. Diagnostic value of LncRNA ROR in differentiating ovarian cancer patients. Clin Lab. 2020;66(7). 10.7754/Clin.Lab.2019.191035 10.7754/Clin.Lab.2019.191035 32658434

[6] Shetty A, Venkatesh T, Kabbekodu SP, Tsutsumi R, Suresh PS. LncRNA-miRNA-mRNA regulatory axes in endometrial cancer: a comprehensive overview. Arch Gynecol Obstet. 2022;306(5):1431–47. 10.1007/s00404-022-06423-5 35182183 10.1007/s00404-022-06423-5

[7] Ghasemi T, Khalaj-Kondori M, Hosseinpour Feizi MA, Asadi P. lncRNA-miRNA-mRNA interaction network for colorectal cancer; an in Silico analysis. Comput Biol Chem. 2020;89:107370. 10.1016/j.compbiolchem.2020.107370 32932199 10.1016/j.compbiolchem.2020.107370

[8] Wang S, Wang Y, Lu J, Wang J. LncRNA LINC00665 promotes ovarian cancer cell proliferation and inhibits apoptosis via targeting miR-181a-5p/FHDC. Appl Biochem Biotechnol. 2022;194(9):3819–32. 10.1007/s12010-022-03943-3 35524876 10.1007/s12010-022-03943-3

[9] Dong B, Li C, Xu X, Wang Y, Li Y, Li X. LncRNA LINC01123 promotes malignancy of ovarian cancer by targeting hsa-miR-516b-5p/VEGFA. Genes Genomics. 2024;46(2):231–9. 10.1007/s13258-023-01440-3 37728844 10.1007/s13258-023-01440-3

[10] Xiong T, Wang Y, Zhang Y, Yuan J, Zhu C, Jiang W. LncRNA AC005224.4/miR-140-3p/SNAI2 regulating axis facilitates the invasion and metastasis of ovarian cancer through epithelial-mesenchymal transition. Chin Med J. 2023;136(9):1098–110. 10.1097/cm9.0000000000002201 36939239 10.1097/CM9.0000000000002201 PMC10228486

[11] Tong L, Wang Y, Ao Y, Sun X. CREB1 induced LncRNA HAS2-AS1 promotes epithelial ovarian cancer proliferation and invasion via the miR-466/RUNX2 axis. Biomed pharmacotherapy = Biomedecine Pharmacotherapie. 2019;115:108891. 10.1016/j.biopha.2019.108891 10.1016/j.biopha.2019.108891 31082772

[12] Ma B, Luo Y, Xu W, Han L, Liu W, Liao T, et al. LINC00886 negatively regulates malignancy in anaplastic thyroid cancer. Endocrinology. 2023;164(4). 10.1210/endocr/bqac204 10.1210/endocr/bqac204 36726346

[13] Lan L, Cao H, Chi W, Meng W, Zhao L, Cui W, et al. Aberrant DNA hypermethylation-silenced LINC00886 gene accelerates malignant progression of laryngeal carcinoma. Pathol Res Pract. 2020;216(4):152877. 10.1016/j.prp.2020.152877 32111441 10.1016/j.prp.2020.152877

[14] Guo J, Zeng H, Li T, Liang X, Peng J, mRNA. LncRNA and circular RNA expression profiles in granulosa cells of infertile women with ovarian endometriosis. Reproductive sciences (Thousand Oaks. Calif). 2022;29(10):2937–46. 10.1007/s43032-022-00966-3 10.1007/s43032-022-00966-3 35799021

[15] Cui M, Liu Y, Cheng L, Li T, Deng Y, Liu D. Research progress on anti-ovarian cancer mechanism of MiRNA regulating tumor microenvironment. Front Immunol. 2022;13:1050917. 10.3389/fimmu.2022.1050917 36439168 10.3389/fimmu.2022.1050917 PMC9684721

[16] Taheri M, Safarzadeh A, Hussen BM, Ghafouri-Fard S, Baniahmad A. LncRNA/miRNA/mRNA network introduces novel biomarkers in prostate cancer. Cells. 2022;11(23). 10.3390/cells11233776 10.3390/cells11233776 PMC9736264 36497036

[17] Tang X, Zeng X, Huang Y, Chen S, Lin F, Yang G, et al. miR-423-5p serves as a diagnostic indicator and inhibits the proliferation and invasion of ovarian cancer. Experimental Therapeutic Med. 2018;15(6):4723–30. 10.3892/etm.2018.6015 10.3892/etm.2018.6015 PMC5960745 29849781

[18] Ferri C, Di Biase A, Bocchetti M, Zappavigna S, Wagner S, Le Vu P, et al. MiR-423-5p prevents MALAT1-mediated proliferation and metastasis in prostate cancer. J Experimental Clin Cancer Research: CR. 2022;41(1):20. 10.1186/s13046-021-02233-w 35016717 10.1186/s13046-021-02233-w PMC8751098

[19] Dong H, Liu Q, Chen C, Lu T, Xu K. LncRNA OGFRP1 promotes angiogenesis and epithelial-mesenchymal transition in colorectal cancer cells through miR-423-5p/CTCF axis. Immunobiology. 2022;227(2):152176. 10.1016/j.imbio.2022.152176 35066433 10.1016/j.imbio.2022.152176

[20] Wang J, Wu M, Chang L, Jin Z, Yang X, Li D, et al. The LncRNA TERC promotes gastric cancer cell proliferation, migration, and invasion by sponging miR-423-5p to regulate SOX12 expression. Annals Translational Med. 2022;10(18):963. 10.21037/atm-22-3545 10.21037/atm-22-3545 PMC9577797 36267723

[21] Xue ST, Zheng B, Cao SQ, Ding JC, Hu GS, Liu W, et al. Long non-coding RNA LINC00680 functions as a CeRNA to promote esophageal squamous cell carcinoma progression through the miR-423-5p/PAK6 axis. Mol Cancer. 2022;21(1):69. 10.1186/s12943-022-01539-3 35255921 10.1186/s12943-022-01539-3 PMC8900330

[22] Yang C, Liu Z, Chang X, Xu W, Gong J, Chai F, et al. NR2F1-AS1 regulated miR-423-5p/SOX12 to promote proliferation and invasion of papillary thyroid carcinoma. J Cell Biochem. 2020;121(2):2009–18. 10.1002/jcb.29435 31692033 10.1002/jcb.29435

[23] Jin Y, Chen L, Li L, Huang G, Huang H, Tang C. SNAI2 promotes the development of ovarian cancer through regulating ferroptosis. Bioengineered. 2022;13(3):6451–63. 10.1080/21655979.2021.2024319 35220872 10.1080/21655979.2021.2024319 PMC8974033

[24] Wang WX, Wilfred BR, Hu Y, Stromberg AJ, Nelson PT. Anti-Argonaute RIP-Chip shows that MiRNA transfections alter global patterns of mRNA recruitment to Microribonucleoprotein complexes. RNA (New York NY). 2010;16(2):394–404. 10.1261/rna.1905910 10.1261/rna.1905910 PMC2811668 20042474

[25] Meier J, Hovestadt V, Zapatka M, Pscherer A, Lichter P, Seiffert M. Genome-wide identification of translationally inhibited and degraded miR-155 targets using RNA-interacting protein-IP. RNA Biol. 2013;10(6):1018–29. 10.4161/rna.24553 23673373 10.4161/rna.24553 PMC4111730

[26] Chen Y, Du H, Bao L, Liu W. LncRNA PVT1 promotes ovarian cancer progression by Silencing miR-214. Cancer Biology Med. 2018;15(3):238–50. 10.20892/j.issn.2095-3941.2017.0174 10.20892/j.issn.2095-3941.2017.0174 PMC6121055 30197791

[27] Tian ML, Li B, Li Y, Fan HW, Du NY, Kang S. LncRNA LINC00261 associates with chemoresistance and clinical prognosis in patients with epithelial ovarian cancer. J Obstet Gynaecol Res. 2024. 10.1111/jog.16077 39390648 10.1111/jog.16077

[28] Dong Z, Yang L, Lu J, Guo Y, Shen S, Liang J, et al. Downregulation of LINC00886 facilitates epithelial-mesenchymal transition through SIRT7/ELF3/miR-144 pathway in esophageal squamous cell carcinoma. Clin Exp Metastasis. 2022;39(4):661–77. 10.1007/s10585-022-10171-w 35616822 10.1007/s10585-022-10171-w

[29] Li L, Ai R, Yuan X, Dong S, Zhao D, Sun X, et al. LINC00886 facilitates hepatocellular carcinoma tumorigenesis by sequestering microRNA-409-3p and microRNA-214-5p. J Hepatocellular Carcinoma. 2023;10:863–81. 10.2147/jhc.S410891 37313303 10.2147/JHC.S410891 PMC10259583

[30] Wang Q, Wang LX, Zhang CY, Bai N, Feng C, Zhang ZM, et al. LncRNA CRNDE promotes cell proliferation, migration and invasion of ovarian cancer via miR-423-5p/FSCN1 axis. Mol Cell Biochem. 2022;477(5):1477–88. 10.1007/s11010-022-04382-8 35166986 10.1007/s11010-022-04382-8

[31] Du W, Feng Z, Sun Q. LncRNA LINC00319 accelerates ovarian cancer progression through miR-423-5p/NACC1 pathway. Biochem Biophys Res Commun. 2018;507(1–4):198–202. 10.1016/j.bbrc.2018.11.006 30442370 10.1016/j.bbrc.2018.11.006

[32] Luo Y, Huang S, Wei J, Zhou H, Wang W, Yang J, et al. Long noncoding RNA LINC01606 protects colon cancer cells from ferroptotic cell death and promotes stemness by SCD1-Wnt/β-catenin-TFE3 feedback loop signalling. Clin Translational Med. 2022;12(4):e752. 10.1002/ctm2.752 10.1002/ctm2.752 PMC9052012 35485210

[33] Jia W, Yu T, An Q, Cao X, Pan H. MicroRNA-423-5p inhibits colon cancer growth by promoting caspase-dependent apoptosis. Experimental Therapeutic Med. 2018;16(2):1225–31. 10.3892/etm.2018.6288 10.3892/etm.2018.6288 PMC6090304 30116373

[34] Lin H, Lin T, Lin J, Yang M, Shen Z, Liu H, et al. Inhibition of miR-423-5p suppressed prostate cancer through targeting GRIM-19. Gene. 2019;688:93–7. 10.1016/j.gene.2018.11.021 30415005 10.1016/j.gene.2018.11.021

[35] Fang J, Jiang G, Mao W, Huang L, Huang C, Wang S, et al. Up-regulation of long noncoding RNA MBNL1-AS1 suppresses breast cancer progression by modulating miR-423-5p/CREBZF axis. Bioengineered. 2022;13(2):3707–23. 10.1080/21655979.2022.2026728 35094653 10.1080/21655979.2022.2026728 PMC8973591

[36] Xu R, Ruan Y, Zhang L, Gu Y, Liu M. Fraxetin suppresses the proliferation, migration, and invasion of ovarian cancer cells by inhibiting the TLR4/STAT3 signaling pathway. Immunopharmacol Immunotoxicol. 2023;45(3):287–94. 10.1080/08923973.2022.2141643 36346016 10.1080/08923973.2022.2141643

[37] Kashani B, Zandi Z, Bashash D, Zaghal A, Momeny M, Poursani EM, et al. Small molecule inhibitor of TLR4 inhibits ovarian cancer cell proliferation: new insight into the anticancer effect of TAK-242 (Resatorvid). Cancer Chemother Pharmacol. 2020;85(1):47–59. 10.1007/s00280-019-03988-y 31786654 10.1007/s00280-019-03988-y

[38] Lv Y, Guo S, Jin L, Wang K, Li Y, Li H, et al. MiR-5195-3p predicts clinical prognosis and represses colorectal cancer progression by targeting TLR4/MyD88 signaling. Cell Div. 2024;19(1):29. 10.1186/s13008-024-00133-x 39390599 10.1186/s13008-024-00133-x PMC11468180

[39] Arkhypov I, Özbay Kurt FG, Bitsch R, Novak D, Petrova V, Lasser S, et al. HSP90α induces immunosuppressive myeloid cells in melanoma via TLR4 signaling. J Immunother Cancer. 2022;10(9). 10.1136/jitc-2022-005551 10.1136/jitc-2022-005551 PMC9486388 36113897

[40] Kang X, Li P, Zhang C, Zhao Y, Hu H, Wen G. The TLR4/ERK/PD–L1 axis May contribute to NSCLC initiation. Int J Oncol. 2020;57(2):456–65. 10.3892/ijo.2020.5068 32468028 10.3892/ijo.2020.5068 PMC7307593

[41] Wang K, Wang J, Liu T, Yu W, Dong N, Zhang C, et al. Morphine-3-glucuronide upregulates PD-L1 expression via TLR4 and promotes the immune escape of non-small cell lung cancer. Cancer Biology Med. 2021;18(1):155–71. 10.20892/j.issn.2095-3941.2020.0442 10.20892/j.issn.2095-3941.2020.0442 PMC7877184 33628591

[42] Youn SE, Jiang F, Won HY, Hong DE, Kang TH, Park YY, et al. PAUF induces migration of human pancreatic cancer cells exclusively via the TLR4/MyD88/NF-κB signaling pathway. Int J Mol Sci. 2022;23(19). 10.3390/ijms231911414 10.3390/ijms231911414 PMC9570212 36232715

[43] Ye C, Yao Z, Wang Y, Zhang C. Asiaticoside promoted ferroptosis and suppressed immune escape in gastric cancer cells by downregulating the Wnt/β-catenin pathway. Int Immunopharmacol. 2024;134:112175. 10.1016/j.intimp.2024.112175 38733821 10.1016/j.intimp.2024.112175

[44] Fang W, Zhou T, Shi H, Yao M, Zhang D, Qian H, et al. Progranulin induces immune escape in breast cancer via up-regulating PD-L1 expression on tumor-associated macrophages (TAMs) and promoting CD8(+) T cell exclusion. J Experimental Clin Cancer Research: CR. 2021;40(1):4. 10.1186/s13046-020-01786-6 10.1186/s13046-020-01786-6 PMC7780622 33390170

