# Supplementary figures and images for: miR-423-5p mediates LINC00886 regulation of ovarian cancer aggressiveness and immune evasion via the TLR4/Myd88/NF-κB/PD-L1 pathway
Source: Hereditas. 2025 Sep 25;162:184. doi: 10.1186/s41065-025-00540-2 (PMC12465903; doi:10.1186/s41065-025-00540-2)

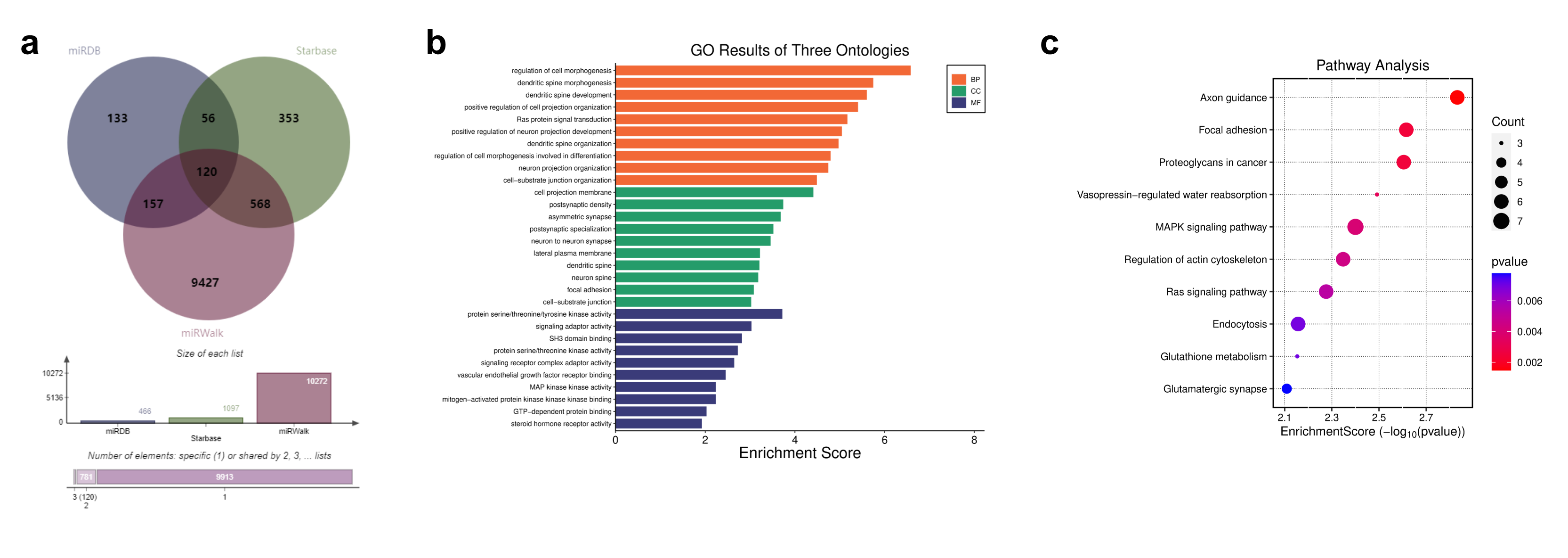

Supplement: Supplementary file 1 — Supplementary Figure 1: Prediction of miR-423-5p target genes and their functional enrichment analysis (a) Venn diagram showing overlapping target genes of miR-423-5p identified by three miRNA target prediction databases (TargetScan, miRDB, and StarBase). Intersection analysis of predictions from these databases yielded 120 unique target genes, which were selected for subsequent functional enrichment analysis. (b) Gene Ontology (GO) functional enrichment analysis of the 120 overlapping target genes, performed using DAVID. The analysis includes three categories: biological process (BP), cellular component (CC), and molecular function (MF). Significantly enriched terms are displayed, including key processes such as “regulation of cell morphogenesis” (BP), “Ras protein signal transduction” (BP), “focal adhesion” (CC), “protein serine/threonine kinase activity” (MF), and “dependent protein binding” (MF), which are associated with cancer initiation and progression. (c) Kyoto Encyclopedia of Genes and Genomes (KEGG) pathway analysis of the 120 target genes, conducted using DAVID. The analysis revealed significant enrichment in pathways relevant to cancer development, including “Focal adhesion,” “Proteoglycans in cancer,” and “MAPK signaling pathway.” For both GO and KEGG analyses, enrichment significance was determined by Fisher’s exact test (P < 0.05), with the top enriched terms/pathways displayed [file 41065_2025_540_MOESM1_ESM.tif]

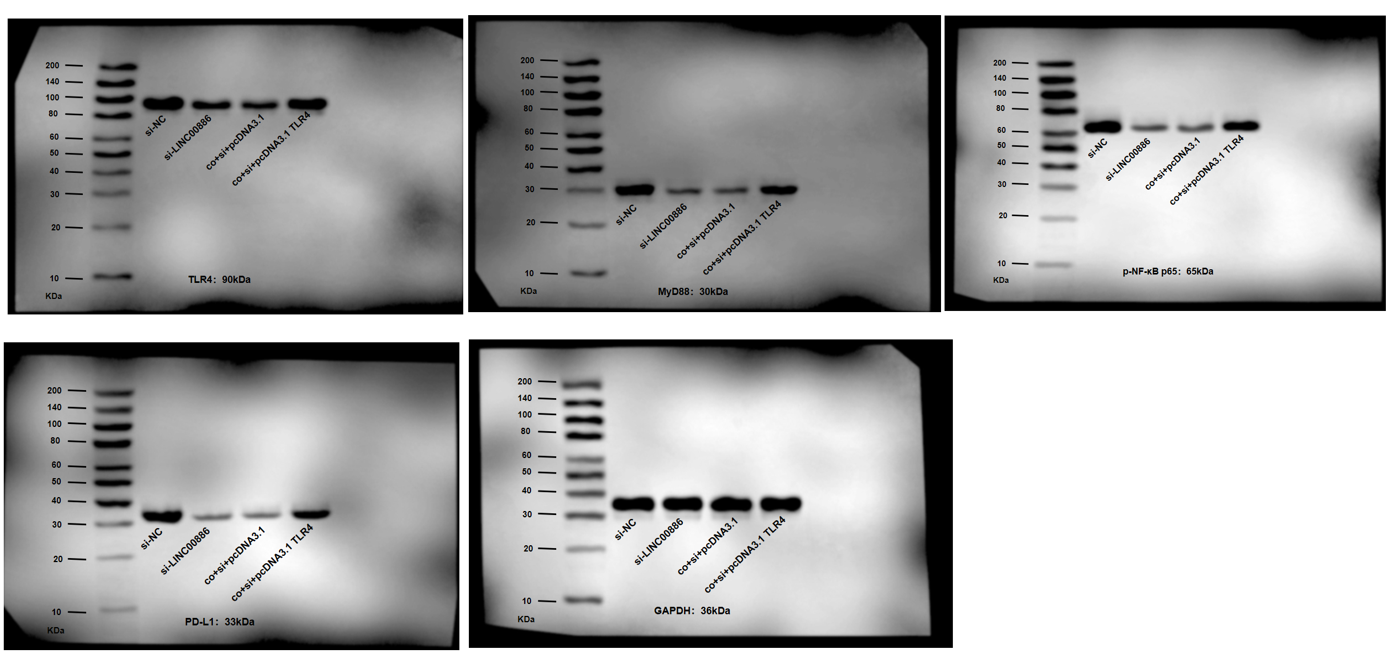

Supplement: Supplementary file 4 — Supplementary Figure 2: Full Western blot gel images for proteins in Fig. 6a-d. Full-length gel images of representative immunoblots from Western blot analysis, corresponding to the quantitative data in Fig. 6a-d. These complete gel images show protein bands for TLR4, Myd88, phosphorylated NF-κB p65 (p-NF-κB p65), PD-L1, and GAPDH (loading control) in COC1 cells under four transfection conditions: (1) si-NC (negative control siRNA); (2) si-LINC00886 (LINC00886 knockdown); (3) si + pcDNA3.1 (co-transfection of si-LINC00886 and empty pcDNA3.1 vector); (4) si-LINC00886 + pcDNA3.1-TLR4 (co-transfection of si-LINC00886 and TLR4 overexpression plasmid) [file 41065_2025_540_MOESM4_ESM.tif]

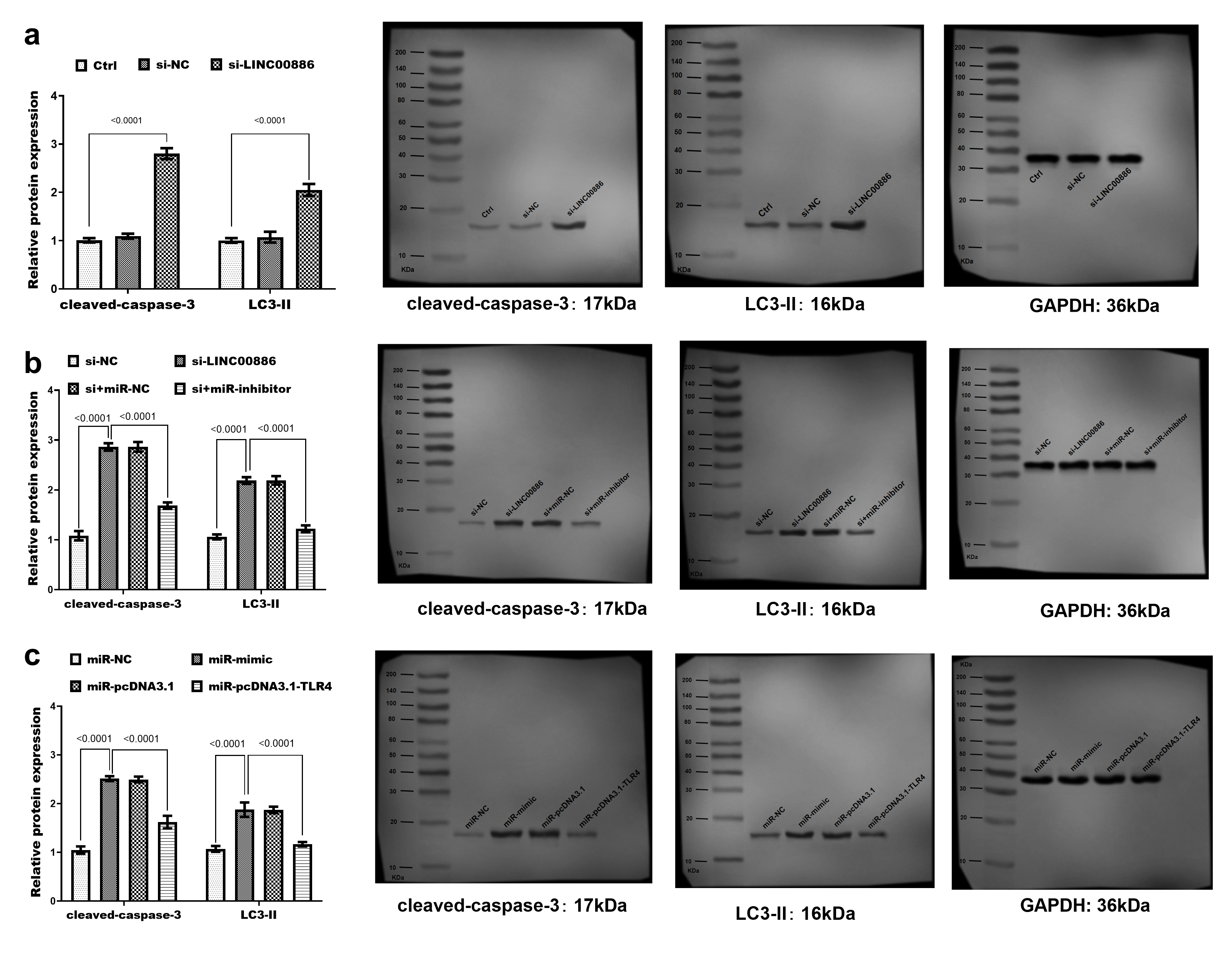

Supplement: Supplementary file 5 — Supplementary Figure 3: Regulation of LINC00886/miR-423-5p/TLR4 axis on autophagy and apoptosis markers. (a) Protein expression levels of autophagy marker LC3-II (autophagosome marker) and apoptotic marker cleaved-caspase-3 in COC1 cells transfected with si-NC (negative control for LINC00886 knockdown) or si-LINC00886 (LINC00886 knockdown), detected by Western blot 48h post-transfection. (b) Protein levels of LC3-II and cleaved-caspase-3 in COC1 cells transfected with: (1) si-NC; (2) si-LINC00886; (3) si + miR-NC (co-knockdown of LINC00886 and negative control for miR-423-5p inhibitor); (4) si + miR-inhibitor (co-knockdown of LINC00886 and miR-423-5p inhibitor), detected by Western blot 48h post-transfection. (c) Protein levels of LC3-II and cleaved-caspase-3 in COC1 cells transfected with: (1) miR-NC (negative control for miR-423-5p mimics); (2) miR-mimic (miR-423-5p overexpression); (3) miR-mimics + pcDNA3.1 (co-transfection of miR-423-5p mimics and empty pcDNA3.1 vector); (4) miR-mimics + pcDNA3.1-TLR4 (co-transfection of miR-423-5p mimics and TLR4 overexpression plasmid), detected by Western blot 48h post-transfection. For all panels: relative protein expression was quantified by densitometry (ImageJ) and normalized to the control group; data are presented as mean ± SD (n = 3). Statistical analysis was performed using one-way ANOVA [file 41065_2025_540_MOESM5_ESM.tif]
