# Supplementary material for: miR-423-5p mediates LINC00886 regulation of ovarian cancer aggressiveness and immune evasion via the TLR4/Myd88/NF-κB/PD-L1 pathway
Source: Hereditas. 2025 Sep 25;162:184. doi: 10.1186/s41065-025-00540-2 (PMC12465903; doi:10.1186/s41065-025-00540-2)
Supplement: Supplementary file 2 — Supplementary Table 1: Primer sequences [file 41065_2025_540_MOESM2_ESM.docx]

Table S1 Primer sequences

| Primer name | | Primer sequence |
| --- | --- | --- |
| LINC00886 | forward | 5'-TGTCTGACTCTGGGCACCTA-3' |
|  | reverse | 5'-GGGCTCCAGTAGAGATATGCC-3' |
| Bax | forward | 5' CGGCGAATTGGAGATGAACTGG3' |
|  | reverse | 5' CTAGCAAAGTAGAAGAGGGCAACC3' |
| Bcl-2 | forward | 5'-CTGAGCAGCGTCTTCAGAGACA-3' |
|  | reverse | 5'-CTGAGCAGCGTCTTCAGAGACA-3' |
| TLR4 | forward | 5'-CAACAAAGGTGGGAATGCTT-3 |
|  | reverse | 5'-TGCCAT TGAAAGCAACTCTG-3' |
| GAPDH | forward | 5'-GGGCATCTTGGGCTACAC-3' |
|  | reverse | 5'-GGTCCAGGGTTTCTTACTCC-3' |
| miR-423-5p | forward | 5'-TGAGGGGCAGAGAGCGA-3' |
|  | reverse | 5'-GTGCGTGTCGTGGAGTCG-3' |
| U6 | forward | 5'-CTCGCTTCGGCAGCACA-3 |
|  | reverse | 5'-AACGCTTCACGAATTTGCGT-3' |

Bcl-2: B-cell lymphoma-2; Bax: BCL-2-associated X protein; TLR4: Toll-like receptor 4
