# Supplementary material for: miR-423-5p mediates LINC00886 regulation of ovarian cancer aggressiveness and immune evasion via the TLR4/Myd88/NF-κB/PD-L1 pathway
Source: Hereditas. 2025 Sep 25;162:184. doi: 10.1186/s41065-025-00540-2 (PMC12465903; doi:10.1186/s41065-025-00540-2)
Supplement: Supplementary file 3 — Supplementary Table 2: Sequence of double luciferase reporter gene [file 41065_2025_540_MOESM3_ESM.docx]

Table S2 Sequence of double luciferase reporter gene

| Name | Sequence |
| --- | --- |
| WT-LINC01094 | 5' AGGGACCUACCCCCAUGACCCAAA**CCCCUC**CCACCAGGCCU 3' |
| miR-423-5p | 3' UUUCAGAGCGAGAGAC**GGGGAG**U 5' |
| MT-LINC01094 | 5' AGGGACCUACCCCCAUGACCCAAA**GGGGAG**CCACCAGGCCU 3' |
|  |  |
| WT-TLR4 | 5' CCACCCCAGGACCUU**GCCCCUC**C 3' |
| miR-494-3p | 3' UUUCAGAGCGAGAGA**CGGGGAG**U 5' |
| MT- TLR4 | 5' CCACCCCAGGACCUU**CGGGGAG**C 3' |

WT: wild type; MT: mutant
